# BMP signaling during craniofacial development: new insights into pathological mechanisms leading to craniofacial anomalies

**DOI:** 10.3389/fphys.2023.1170511

**Published:** 2023-05-18

**Authors:** Hiroki Ueharu, Yuji Mishina

**Affiliations:** Department of Biologic and Materials & Prosthodontics, University of Michigan School of Dentistry, Ann Arbor, MI, United States

**Keywords:** craniofacial development, BMP, transgenic animal, orofacial cleft, craniosynostosis

## Abstract

Cranial neural crest cells (NCCs) are the origin of the anterior part of the face and the head. Cranial NCCs are multipotent cells giving rise to bones, cartilage, adipose-tissues in the face, and neural cells, melanocytes, and others. The behavior of cranial NCCs (proliferation, cell death, migration, differentiation, and cell fate specification) are well regulated by several signaling pathways; abnormalities in their behavior are often reported as causative reasons for craniofacial anomalies (CFAs), which occur in 1 in 100 newborns in the United States. Understanding the pathological mechanisms of CFAs would facilitate strategies for identifying, preventing, and treating CFAs. Bone morphogenetic protein (BMP) signaling plays a pleiotropic role in many cellular processes during embryonic development. We and others have reported that abnormalities in BMP signaling in cranial NCCs develop CFAs in mice. Abnormal levels of BMP signaling cause miscorrelation with other signaling pathways such as Wnt signaling and FGF signaling, which mutations in the signaling pathways are known to develop CFAs in mice and humans. Recent Genome-Wide Association Studies and exome sequencing demonstrated that some patients with CFAs presented single nucleotide polymorphisms (SNPs), missense mutations, and duplication of genes related to BMP signaling activities, suggesting that defects in abnormal BMP signaling in human embryos develop CFAs. There are still a few cases of BMP-related patients with CFAs. One speculation is that human embryos with mutations in coding regions of BMP-related genes undergo embryonic lethality before developing the craniofacial region as well as mice development; however, no reports are available that show embryonic lethality caused by BMP mutations in humans. In this review, we will summarize the recent advances in the understanding of BMP signaling during craniofacial development in mice and describe how we can translate the knowledge from the transgenic mice to CFAs in humans.

## Introduction

### Craniofacial anomalies (CFAs)

Craniofacial anomalies (CFAs) are birth defects affecting the shape of the head and the face. The incidence is approximately 1 in 100 newborns (Twigg and Wilkie, 2015; Sakai and Trainor, 2016; Wei et al., 2016; Kitami et al., 2018). Orofacial cleft (1:700) and craniosynostosis (1:2000) are the most common CFAs (Morriss-Kay and Wilkie, 2005; Nasreddine et al., 2021). Approximately 70% of patients with orofacial cleft and 85% of patients with craniosynostosis are non-syndromic, with no other abnormalities in the patients, and most have no other affected family members (Timberlake et al., 2016; Howe et al., 2018). Therefore, it has been unclear whether genetic or non-genetic reasons cause that, and caused by single gene mutation or combined effect of several genes. The current primary therapeutic option for orofacial cleft and craniosynostosis is invasive surgeries, which decrease their quality of life (Esparza and Hinojosa, 2008; Seruya et al., 2011; Shkoukani et al., 2014; Opriş et al., 2022; Spazzapan et al., 2022). Therefore, defining the molecular and cellular pathogenesis of CFAs could facilitate the strategies for the early identification, prevention, and treatment of these developmental diseases. Cranial neural crest cells (NCCs) are the origin of the face and the anterior part of the head (Mishina and Snider, 2014). Cranial NCCs are multipotent cells giving rise to osteoblasts, chondrocytes, adipocytes, melanocytes, odontoblasts, neural cells, glia, pituitary hormone-producing cells and others (Le Douarin et al., 2004; Ishii et al., 2012; Ueharu et al., 2017; Srinivasan and Toh, 2019). Defects of migration, proliferation, cell death, differentiation, and cell fate specification in cranial NCCs are often reported as causative reasons for CFAs (Yu et al., 2005; Teng et al., 2008; Gu et al., 2014). Thus, understanding the regulation mechanisms of cranial NCCs would provide new therapeutic options for CFAs. Two transgenic mice, *Wnt1-Cre* mice and *P0-Cre* mice, are largely used for tracing NCCs and NC-specific activation or deletion of targeted genes by the Cre-LoxP system (Danielian et al., 1998; Yamauchi et al., 1999). Both transgenic mice label cranial NCCs in a similar manner, but due to the small differences in the expression patterns, resulting mutant mice sometimes show phenotype differences between *Wnt1-Cre* mice and *P0-Cre* mice (Wang et al., 2011; Kulkarni et al., 2018; Zhang et al., 2022; Ueharu et al., 2023b). Detailed comparison between 2 Cre transgenes using *R26-LacZ* reporter mice revealed that *Wnt1-Cre* activity initially found at the midbrain region while that of *P0-Cre* is found at the hindbrain region (Chen et al., 2017). *Wnt1* (not *Wnt1-Cre*) expression is more abundant in premigratory neural crest cells that post-migratory cells (Echelard et al., 1994), while *P0-Cre* proteins present at the 19 somite stage (Chen et al., 2017). Expression of endogenous *P0* gene during neural crest development is not available. Taken together, these differences may result similar but unique phenotypes.

## Bone morphogenetic proteins (BMPs)

**FIGURE 1:**
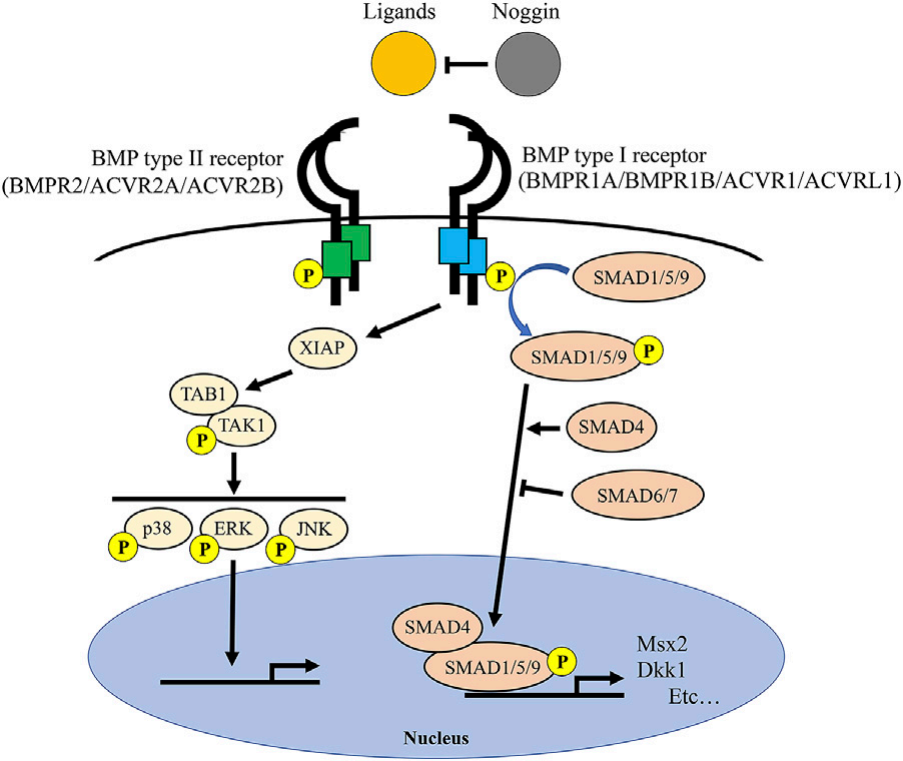
BMP signaling and BMP-related genes. Once BMP ligands bind to BMP receptor type 2 (BMPR2/ACVR2A/ACVR2B) and BMP receptor type 1 (BMPR1A/BMPR1B/ACVR1/ACVRL1) complexes, type 1 receptors are phosphorylated by type 2 receptors. Subsequently, BMP type 1 receptors phosphorylate SMAD1/5/9 proteins that transduce signals to the nucleus with SMAD4 protein to alter expressions of downstream target genes (e.g., *Msx2* and *Dkk1*). Inhibitory SMAD6 and SMAD7 prevent the phosphorylation and thus nuclear transition of SMAD1/5/9 proteins. Noggin is an extracellular antagonist for the BMP ligands to suppress BMP signaling.

Bone morphogenetic proteins (BMPs) are members of the Transforming Growth Factor Beta superfamily. Upon BMP ligands (e.g., BMP2, BMP4, BMP7) binding, BMP receptors form hetero-multimers consisting of BMP type II receptors (BMPR2 and ACVR2A and ACVR2B) and BMP type I receptors (BMPR1A, BMPR1B, ACVR1, and ACVRL1) then transduce the signaling through phosphorylation of SMAD1/5/9 (SMAD-dependent pathway) or TAK1/p38 MAP kinases (SMAD-independent pathway) (Wu et al., 2016; Gomez-Puerto et al., 2019). The signaling level of BMPs is well regulated by a BMP antagonist Noggin and inhibitory SMAD6/7 (Figure 1).

Bone morphogenetic proteins (BMPs) were originally discovered as bone inducers (Urist, 1965). Interestingly, recent reports published by our group and others demonstrated that BMP signaling plays pleiotropic roles in embryogenesis, including craniofacial development, by regulating proliferation, cell death, differentiation, and cell fate specification, in addition to osteogenesis (Komatsu et al., 2013; Graf et al., 2016; Grafe et al., 2018; Yang et al., 2021a). Many studies have shown that loss of function mutation or gain of function mutation of BMPs, BMP receptors, and downstream target genes for BMP signaling in mice develops CFAs; however, only a few case reports of human patients with BMP mutations are available. In this review, we will describe that transgenic mice targeted for BMP signaling and BMP-downstream genes that develop CFAs. We will further discuss a potential reason of the discrepancy in why it is rare to see human CFAs patients with BMP mutations while many transgenic mice develop CFAs.

## Orofacial cleft

Orofacial cleft (cleft lip, cleft palate, and midfacial cleft) is the most common CFAs that show splitting lip and/or splitting shelves of the mouth (palate) (Shkoukani et al., 2014). Because of the abnormal structures, patients with an orofacial cleft frequently have difficulties feeding, swallowing, and breathing (Warren et al., 1992; Reid, 2004; Duarte et al., 2016). Serial invasive surgeries after birth are the only therapeutic options (Srinivasan and Toh, 2019), which are burdens for the patients and increase medical expenses; thus understanding molecular mechanisms of pathological craniofacial development could facilitate the strategies for early identification and prevention of orofacial cleft, which will provide us novel therapeutic options such as taking a drug to the pregnant mothers to prevent developing orofacial cleft, in addition to surgeries.

The Pierre-Robin sequence is a birth defect characterized by an underdeveloped lower jaw (Hsieh and Woo, 2019). The underdeveloped lower jaw results in bringing the tongue back and preventing closure of the palate. As a result of the smaller jaw, approximately 85% of Patients with the Pierre-Robin sequence develop a cleft palate (Hsieh and Woo, 2019). *Bmp7*-deficient mice (*Bmp7* ^Δ/Δ^ mice) develop cleft palates with the shorter Meckel’s cartilage (Kouskoura et al., 2013). Their *ex vivo* culture of palatal shelves showed that palatal shelves from *Bmp7* ^Δ/Δ^ mice retain the ability to fuse when placed in close proximity (Kouskoura et al., 2013). This fact may suggest that the shorter Meckel’s cartilage by *Bmp7* deletion could develop a cleft palate by mimicking the Pierre-Robin sequence. However, *ex vivo* culture of the mutant embryos after removal of the mandibular, the tongue and the brain show poor shelf elevation and failure to fuse, suggesting directing involvement of BMP7 function in palatial shelf elevation (Kouskoura et al., 2013). Palatal mesenchyme-specific Noggin expressing mice (*Osr2-Cre;pMes-Noggin* mice) develop cleft palate with lower proliferation and suppressed osteogenic condensation at the palate (Li et al., 2021). On the other hand, neural crest (NC)-specific deletion of *Bmp2* in mice (*Wnt1-Cre;Bmp2* ^*fl/fl*^ mice) develops cleft palate as a consequence of the failure of tongue descent (Chen et al., 2019). *Wnt1-Cre;Bmp2* ^*fl/fl*^ mice showed lower cell proliferation in the mandibular and the Meckel’s cartilage, but not in the anterior and posterior palate, than that in control mice, along with the failure of the tongue descent. These studies suggest that downregulation of BMP signaling causes lower proliferation and suppressed differentiation in the craniofacial tissues that causes miscoordinations of craniofacial development, resulting in developing orofacial cleft, but its etiology may not be same as that of the Pierre-Robin Sequence.

On the other hand, NC-specific expression of constitutively activated *Bmpr1a* (*caBmpr1a*, the kinase activity is ligand-independent due to the Q233D mutation) in mice (*Wnt1-Cre;pMes-caBmpr1a* mice) also develop cleft lip and cleft palate. *Wnt1-Cre;pMes-caBmpr1a* mice showed reduced proliferation and ectopic cartilage formation at the palatal mesenchyme (Li et al., 2013). NC-specific deletion of *Tak1* in mice (*Wnt1-Cre;Tak1* ^*fl/fl*^ mice) develops cleft palate association with a higher level of fibroblast growth factor (FGF) signaling that is a known causative reason for Apert syndrome, a subset of patients with the syndrome develops cleft palate (Song et al., 2013; Willie et al., 2022). We found that NC-specific expression of *caBmpr1a* with deletion of *Tak1* in mice (*P0-Cre;caBmpr1a* ^*fl/+*^ *;Tak1* ^*fl/fl*^ mice) develops cleft palate while *P0-Cre;caBmpr1a* ^*fl/+*^ mice and *P0-Cre;Tak1* ^*fl/fl*^ mice did not develop a cleft palate. (Liu et al., 2018). Although the phenotype differences between us and other laboratories might be developed by the difference of *Wnt1-Cre* mice and *P0-Cre* mice, it is reasonable to speculate that unbalance between the SMAD-dependent and SMAD-independent pathways in cranial NCCs may be a reason for an orofacial cleft in *P0-Cre;caBmpr1a* ^*fl/+*^ *;Tak1* ^*fl/fl*^ mice. We recently reported that two transgenic mouse lines expressing constitutively activated *Acvr1* (*caAcvr1*, *Acvr1* with the Q207D mutation) in NCCs in mice (*P0-Cre;caAcvr1-L35 line* and *P0-Cre;caAcvr1-A11 line*) develops midfacial defects including orofacial cleft (Yang et al., 2021a; Yang et al., 2021b). Interestingly, the facial phenotypes between *P0-Cre;caAcvr1-L35 line* and *P0-Cre;caAcvr1-A11 line* are different. There are still questions why three transgenic mice, i.e., *caBmpr1a* mice, *caAcvr1-L35* mice, and *caAcvr1-A11* mice crossed with *P0-Cre* mice, showed different phenotypes. We found that the phosphorylation level of SMAD1/5/9 differs between *caAcvr1-L35* mice and *caAcvr1-A11* mice (Yang et al., 2021b), which may be a reason for similar but distinct phenotypes between these two lines. Another possibility is that because these mice have been generated through random transgenesis, their expression patterns may be different depending on the genomic locus where the transgenic constructs are integrated. The third possibility is intrinsic differences between type 1 receptors. For example, ACVR1 is known to bind to activins without signal transduction, while BMPR1A does not bind to activins (Alessi Wolken et al., 2018). Phosphorylation of Smad1/5/9 is the common downstream event but there may be unique functions of each receptor for signal transduction, which is poorly understood.

Taken together, craniofacial tissues coordinate to develop the craniofacial region, and abnormal SMAD-dependent pathways and SMAD-independent pathways disrupt the orchestration, causing CFAs such as orofacial cleft.

## Craniosynostosis

Craniosynostosis is another common CFAs characterized by abnormal shapes of the skull and the face caused by premature fusions of cranial sutures. Cranial sutures consist of mesenchymal tissues housing stem/progenitor cells to support the growth of infant’s bones. Premature fusion of cranial sutures results in an imbalance of growth between the skull and brain leading to an increase of intracranial pressure and may secondarily develop neurologic issues such as deafness (Swanson and Mishina, 2021). It has been thought that excess osteogenic differentiation of suture mesenchymal cells causes craniosynostosis. As the name implies, BMP signaling prompts osteogenic differentiation. Many scientists focused on the relation between craniosynostosis and BMP signaling; however, recent studies have shown that BMP signaling develops craniosynostosis by disruptions of cranial NCCs and suture stem cells, in addition to prompt osteogenic differentiation of osteoblasts.

In 1993, a gain of function mutation of MSX2, a downstream target of BMP signaling, was found in human patients with the Boston-type craniosynostosis (Jabs et al., 1993). Higher Msx2 expression at the middle of the sagittal suture in mice developed overgrown parietal bones (Liu et al., 1999). Interestingly, NC-specific deletions (cKO) of three out of four alleles in Msx1/Msx2 in mice (*Wnt1-Cre;Msx1* ^*cKO/+*^ *;Msx2* ^*cKO/cKO*^ or *Wnt1-Cre;Msx1* ^*cKO/cKO*^ *;Msx2* ^*cKO/+*^) develop large calvarial defects in the frontal bones as expected; however, NC-specific deletions of all four alleles in Msx1/Msx2 in mice (*Wnt1-Cre;Msx1* ^*cKO/cKO*^ *;Msx2* ^*cKO/cKO*^) develop ectopic bones at the calvaria (Roybal et al., 2010). Tamoxifen-inducible deletions of Msx1/Msx2 in mice (Cagg-CreER™; *Msx1* ^*cKO/cKO*^ *;Msx2* ^*cKO/cKO*^ mice) demonstrated that Msx1 and Msx2 at E10.5 to E11.5 are required to suppress the ectopic bone formation while those at E11.5 to E12.5 are required to prompt calvarial formation (Roybal et al., 2010). These results suggest that MSX proteins during calvarial development control two distinct biological processes; one is to suppress the cell fate of neural crest cells towards osteogenic differentiation and the other is to prompt osteogenic differentiation once the progenitor cells commit to osteogenic lineage.

An antagonist of BMP signaling *Noggin* is expressed in the patent sutures (Warren et al., 2003). Elevation of FGF signaling, a causative reason for some craniosynostosis such as Crouzon syndrome and Apert syndrome, suppressed *Noggin* expression in the coronal suture in mice (Warren et al., 2003). Inhibition of *Smad7* expression in the calvaria results in premature fusion of the coronal suture (Zhou et al., 2014). Smad6 and Smad7 are known as inhibitory Smads inhibiting signaling pathways of the TGFβ superfamily (Massagué and Chen, 2000). Protein levels of FGF10 and phosphorylated ERK1/2, a transducer of FGF signaling, were elevated by *siSmad7*-treated suture cells, in which TGFβ signaling level was augmented while BMP signaling level was not examined in this experiment (Zhou et al., 2014). We previously reported that *P0-Cre;caBmpr1a* mice showed elevation of FGF signaling; however, unlikely the case of Crouzon syndrome, the elevated FGF signaling does not directly involve in craniosynostosis (Komatsu et al., 2013). Suggestively, it is reported that during calvarial development BMP signaling negatively regulates levels of FGF signaling (Maruyama et al., 2010; Maruyama et al., 2017). When BMP signaling was enhanced in *Axin2* expressing cells, resulting mice show premature suture fusion with ectopic cartilage, but FGF signaling is suppressed (Maruyama et al., 2017). Taken together, these suggest that augmented BMP signaling is a primary reason for craniosynostosis in these mouse models. However, there is a sharp contrast about the levels of FGF signaling between two models, suggesting that mechanisms of how BMP signaling regulates FGF signaling is developmental stage and cell type-specific manner. In our *P0-Cre;caBmpr1a* mice, an elevation of BMP signaling in neural crest cells starts around E8.0-E8.5 (Komatsu et al., 2013; Ueharu et al., 2023b); however, Maruyama et al. designed to elevate BMP signaling in *Axin2*-expressing suture stem cells during late embryonic stage to newborn stage by Doxycycline, and they observed elevated BMP signaling activity at postnatal day 3 (Maruyama et al., 2017). The differences in cell type and developmental stage between the two animal models may generate different outcomes in FGF signaling.

**FIGURE 2:**
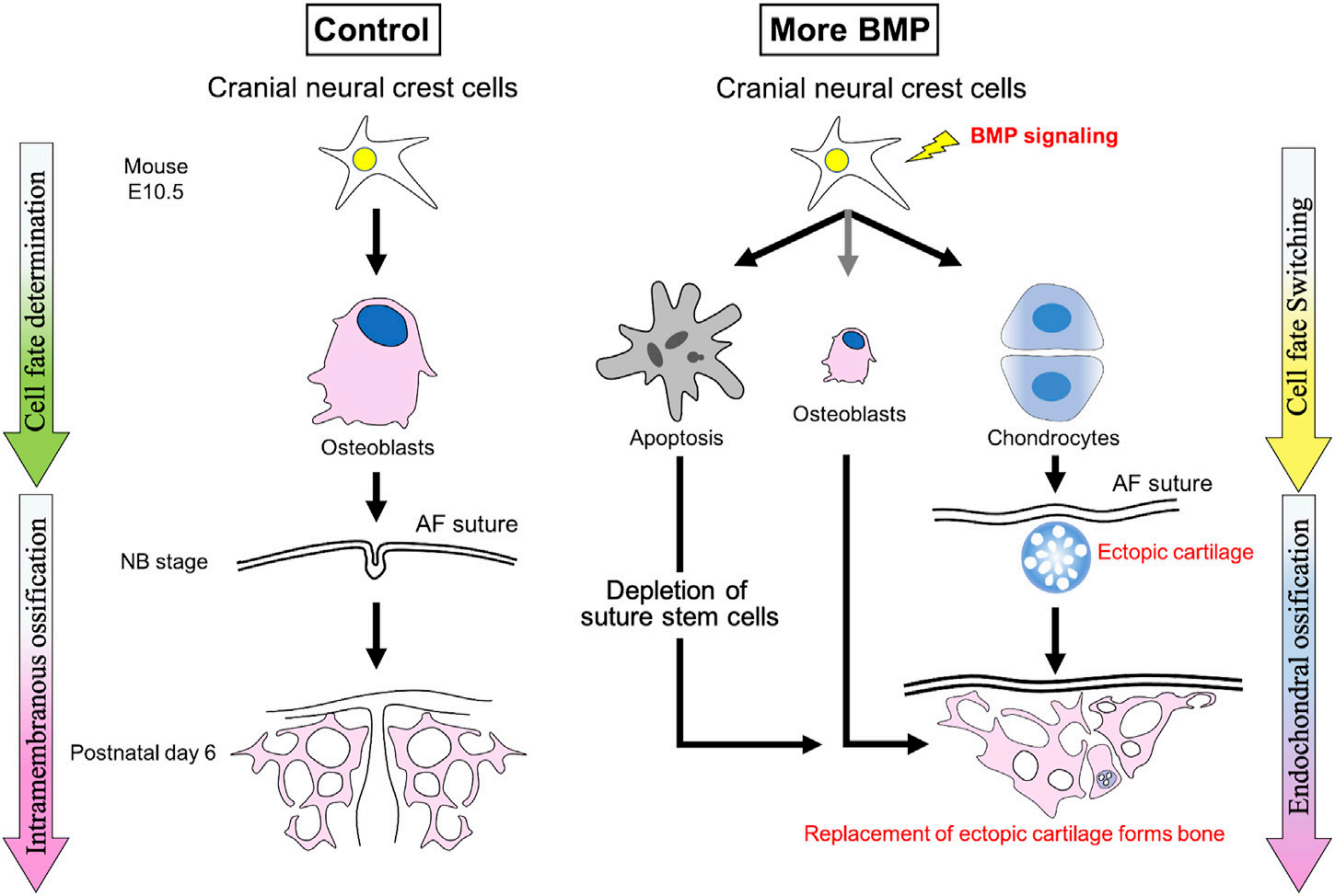
Augmentation of BMP signaling in cranial neural crest cells develops craniosynostosis through endochondral ossification due to cell fate switching towards chondrogenic fate. Enhanced BMP signaling alters cell fate of cranial neural crest cells towards chondrogenic fate at early embryonic stages along with excess cell death leading to ectopic cartilage formation in the sutures, where cause premature suture fusion. These data suggest that the premature fusion of cranial sutures is caused by endochondral ossification prompted by ectopically formed cartilage.

We reported that NC-specific expression of *caBmpr1a* in mice (*P0-Cre;caBmpr1a* mice) develops premature fusion of the anterior frontal suture and the naso-premaxillary suture, which leads to craniosynostosis (Komatsu et al., 2013; Pan et al., 2017; Kramer et al., 2018; Liu et al., 2018; Ueharu et al., 2023b). We found elevated cell death in cranial NCCs in *P0-Cre;caBmpr1a* mice, and inhibition of p53-induced cell death partially rescued premature suture fusion (Komatsu et al., 2013; Hayano et al., 2015; Ueharu et al., 2023a; Ueharu et al., 2023b). Of note, ectopic cartilage is developed only in the sutures which prematurely fused, and during the fusion process, the ectopic cartilage is replaced into bone nodules (Ueharu et al., 2023b). It is reported that global knockout of *Axin2* with heterozygous *Fgfr1* develops premature suture fusion in the presence of ectopic cartilage in sutures (Maruyama et al., 2010). Together with a follow up report, it is suggested that an imbalance between BMP and FGF signaling may alter cell fate of cranial suture mesenchymal cells to develop ectopic cartilage leading to premature fusion of cranial sutures (Maruyama et al., 2017). Thus, we propose that augmentation of BMP signaling in cranial NCCs prompts them towards chondrogenic fate and that results in premature suture fusion through endochondral ossification (Figure 2). In the future, it is an essential effort to identify how cell death and ectopic cartilage formation cooperatively or independently causes craniosynostosis. Interestingly, the premature suture fusion patterns between *P0-Cre;caBmpr1a* mice and *Wnt1-Cre;caBmpr1a* mice are different, which the anterior frontal suture commonly causes premature fusion in both *P0-Cre;caBmpr1a* mice and *Wnt1-Cre;caBmpr1a* mice while the naso-premaxillary suture causes premature fusion only in *P0-Cre;caBmpr1a* mice (Ueharu et al., 2023b). As we discussed, *P0-Cre* mice and *Wnt1-Cre* mice showed similar but not identical recombination patterns. Therefore, utilizing the two transgenic mice could facilitate to identify the pathological mechanisms for craniosynostosis by BMP signaling. On the other hand, *Bmpr1a* cKO mice (*Axin2* ^*Cre*−*Dox*^ *;Bmpr1a* ^*fl/fl*^) develop craniosynostosis (Maruyama et al., 2021). Self-renewal and osteogenic capability of suture mesenchymal stem cells were dramatically reduced in *Bmpr1a* cKO mice. These results suggest that defects of stem cells in sutures are one of the reasons for craniosynostosis rather than excess osteogenic differentiation by BMP signaling. The insights are letting us shift the strategies to the next step, which is how the stemness of suture stem cells is controlled, and whether we can control their stemness in a timely manner by genetic and epigenetic methods.

Taken together, both loss of and gain of function mutations of BMP signaling develop craniosynostosis and orofacial cleft. These results suggest that BMP signaling plays a critical role in calvarial development depending on stages of development by fine-tuning in proliferation, cell death, differentiation, and cell fate specification of cranial NCCs.

## Human cases

We described that many of BMP signaling-targeted transgenic mice develop CFAs. However, there are few reports regarding BMP-related CFAs in human. Here, we describe human cases of BMP-related CFAs and discuss how knowledge from animal models can help understanding etiology of BMP-related CFAs in human.

Duplication of human chromosome 10 q22.3q23.2, which includes BMPR1A, develops a hypertelorism (van Bon et al., 2011). Micro deletion of 20p12.3, including BMP2, develops cleft lip and palate (Sahoo et al., 2011). BMP4 polymorphisms are found in patients with cleft lips w/wo cleft palate (Li et al., 2017). Those reports may suggest that defects in BMP signaling develop CFAs in humans. In cases of mice, global loss of function mutations and gain of function mutations in BMP ligands, BMP receptors, and BMP-related genes causes embryonic lethal at the early embryonic stage (Mishina et al., 1995; Winnier et al., 1995; Zhang and Bradley, 1996; Mishina et al., 1999). It is reasonable to speculate that a significant change in BMP signaling activity may also cause lethality in human subjects. Recently, it has been reported that homozygous missense mutation of BMPR1A (results in amino acid substitution of BMPR1A^R406L^) develops brachycephaly with unilateral coronal craniosynostosis in humans with a slight elevation of phospho-SMAD levels (Russell et al., 2019). Mutations in ACVRL1 (ACVRL1^V228I^, kinase domain) and ACVR2A (ACVR2A^T63A^, BMP7 binding site) are found in patients with lambdoid craniosynostosis (Timberlake et al., 2022). Despite a lack of experimental evidence, it is possible to speculate that these two mutations alter levels of BMP signaling. These results suggest that slight elevation or slight suppression of BMP signaling in humans develop CFAs otherwise they cause embryonic lethal at an early embryonic stage.

Recent reports focusing on patients with non-syndromic craniosynostosis showed that single nucleotide polymorphisms (SNPs) were found in a Smad6 exon and a putative enhancer region of BMP2, and frameshift mutations in Smad6 were found (Justice et al., 2012; Komatsu and Mishina, 2016; Timberlake et al., 2016; Timberlake et al., 2018). SMAD6 is an inhibitory SMAD, that inhibits the phosphorylation of SMAD1/5/9 (Estrada et al., 2011). As discussed above, patients with unilateral coronal craniosynostosis slightly elevated the phospho-SMAD1/5/9 level (Russell et al., 2019). Based on the knowledge from animal studies, it is reasonable to speculate that mutations in regulatory sequences may slightly alter levels of wild-type proteins, which may lead to pathologic conditions. From that point of view, the fact that a limited portion of human subjects with the SNPs in the putative enhancer region of BMP2 develops CFA while all patients who also have mutations in a SMAD6 exon develop craniosynostosis eloquently demonstrate small increase of BMP signaling is critically involved in etiology of CFAs in human.

There are still uninvaded niches, especially, why missense or nonsense mutations in genes related to BMP signaling are rarely found in patients with CFAs. In the future, it is an essential effort to analyze BMP mutations in preterm and stillbirth babies. It is also essential to identify gene mutations in patients with non-syndromic CFAs. Genome-wide association studies (GWAS) and exome sequencing are powerful tools to determine whether they have mutations in BMP ligands, BMP receptors, Smads, noncanonical signaling of BMP signaling, and downstream target genes of BMP signaling, especially identifying SNPs in regulatory regions. We also need to examine whether these mutations change signaling levels of BMP signaling by testing the phosphorylation of SMAD1/5/9 and non-SMAD signaling levels, which is less understood at this moment. These efforts could provide novel therapeutic options for non-syndromic patients with CFAs.
